# A Metalloproteinase Secreted by *Streptococcus pneumoniae* Removes Membrane Mucin MUC16 from the Epithelial Glycocalyx Barrier

**DOI:** 10.1371/journal.pone.0032418

**Published:** 2012-03-07

**Authors:** Bharathi Govindarajan, Balaraj B. Menon, Sandra Spurr-Michaud, Komal Rastogi, Michael S. Gilmore, Pablo Argüeso, Ilene K. Gipson

**Affiliations:** Schepens Eye Research Institute, Massachusetts Eye and Ear, Harvard Medical School, Boston, Massachusetts, United States of America; Instituto Butantan, Brazil

## Abstract

The majority of bacterial infections occur across wet-surfaced mucosal epithelia, including those that cover the eye, respiratory tract, gastrointestinal tract and genitourinary tract. The apical surface of all these mucosal epithelia is covered by a heavily glycosylated glycocalyx, a major component of which are membrane-associated mucins (MAMs). MAMs form a barrier that serves as one of the first lines of defense against invading bacteria. While opportunistic bacteria rely on pre-existing defects or wounds to gain entry to epithelia, non opportunistic bacteria, especially the epidemic disease-causing ones, gain access to epithelial cells without evidence of predisposing injury. The molecular mechanisms employed by these non opportunistic pathogens to breach the MAM barrier remain unknown. To test the hypothesis that disease-causing non opportunistic bacteria gain access to the epithelium by removal of MAMs, corneal, conjunctival, and tracheobronchial epithelial cells, cultured to differentiate to express the MAMs, MUCs 1, 4, and 16, were exposed to a non encapsulated, non typeable strain of *Streptococcus pneumoniae* (SP168), which causes epidemic conjunctivitis. The ability of strain SP168 to induce MAM ectodomain release from epithelia was compared to that of other strains of *S. pneumoniae*, as well as the opportunistic pathogen *Staphylococcus aureus*. The experiments reported herein demonstrate that the epidemic disease-causing *S. pneumoniae* species secretes a metalloproteinase, ZmpC, which selectively induces ectodomain shedding of the MAM MUC16. Furthermore, ZmpC-induced removal of MUC16 from the epithelium leads to loss of the glycocalyx barrier function and enhanced internalization of the bacterium. These data suggest that removal of MAMs by bacterial enzymes may be an important virulence mechanism employed by disease-causing non opportunistic bacteria to gain access to epithelial cells to cause infection.

## Introduction

A characteristic feature of all wet-surfaced mucosal epithelia of the body is the presence of mucins on their apical surface that separate underlying epithelial cells from the external milieu. Mucins are a family of high molecular weight, O-glycosylated proteins that can be classified as either secreted or membrane-associated based on their molecular structure. Secreted mucins, produced by goblet cells, occupy the uppermost, extensively hydrated mucus coating that is moved over the epithelium and functions primarily to sweep away trapped foreign material. On the other hand, membrane-associated mucins (MAMs) anchored to the apical epithelial cell membrane by their single transmembrane domain [1] form an extensively O-glycosyated glycocalyx that serves as a barrier [2], [3] and prevents commensal microbes from adhering to the epithelium [3]. In addition, the short cytoplasmic tail of some MAMs has been demonstrated to be involved in signal transduction pathways [4], [5].

The expression, distribution, biophysical properties, and functions of MAMs vary depending on the type of host mucosal epithelium in question. In humans, MAMs MUCs 1 and 4 are expressed universally across mucosal epithelia; MUCs 3A, 3B, 11, and 12 are expressed by gut epithelia; and MUC16 is expressed by ocular surface, respiratory tract, and the female reproductive tract epithelia [6]. MUC16 is the largest known MAM, with a molecular weight of >200 kDa [5]. When extended, its extracellular domain has been estimated to protrude up to 500 nm from the epithelial surface [7], [8].

Although there has been speculation that MAMs play an important role in host defense against infectious agents by providing a barrier against invading pathogens, the only data available comes from studies of *Muc1^−/−^* mice (mucins in humans are designated as MUC and those in mice as Muc). *Muc1^−/−^* mice were shown to be at increased risk of chronic infection as well as inflammation of the reproductive tract [9] and the ocular surface [10] in comparison to wild type mice. Later studies showed that the intestinal pathogen *Campylobacter jejuni* is capable of enhanced colonization, rapid passage across the gastrointestinal barrier, and induction of epithelial cell damage in *Muc1^−/−^* mice [11]. Similarly, Muc1 was found to limit acute and chronic colonization by the gastritis-causing pathogen *Helicobacter pylori* [12].

Little information is available on the role of MUC16 in host defense. Recent data demonstrated that MUC16 expressed at the human ocular surface possesses barrier functions [3], [13], [14]. Specifically, MUC16 prevents adhesion of the opportunistic pathogen *Staphylococcus aureus* in human corneal epithelial (HCLE) cells [3]. Observations of the anti-adhesive, barrier properties of MUC16 at the epithelial surface raise an important question about the ability of non opportunistic pathogens, especially the epidemic disease-causing ones, to breach the MAM glycocalyx barrier and trigger infection. To address this question and to characterize mechanisms employed by such pathogens, the ability of an epidemic conjunctivitis-causing, non encapsulated, non typeable strain of *S. pneumoniae* and other strains of *S. pneumoniae*, as well as the opportunistic pathogen *S. aureus* to manipulate the MAM barrier was compared. Data presented in this manuscript show that the epidemic disease-causing species of *S. pneumoniae* secretes a zinc metalloproteinase, ZmpC, that selectively induces ectodomain shedding of MUC16 and that the removal of MUC16 compromises MAM glycocalyx barrier function, enhancing internalization of the bacterium.

## Results

### Growth culture filtrates of *S. pneumoniae* strain SP168 and serotype 11A induce MUC16 shedding from epithelial cells

**Figure 1:**
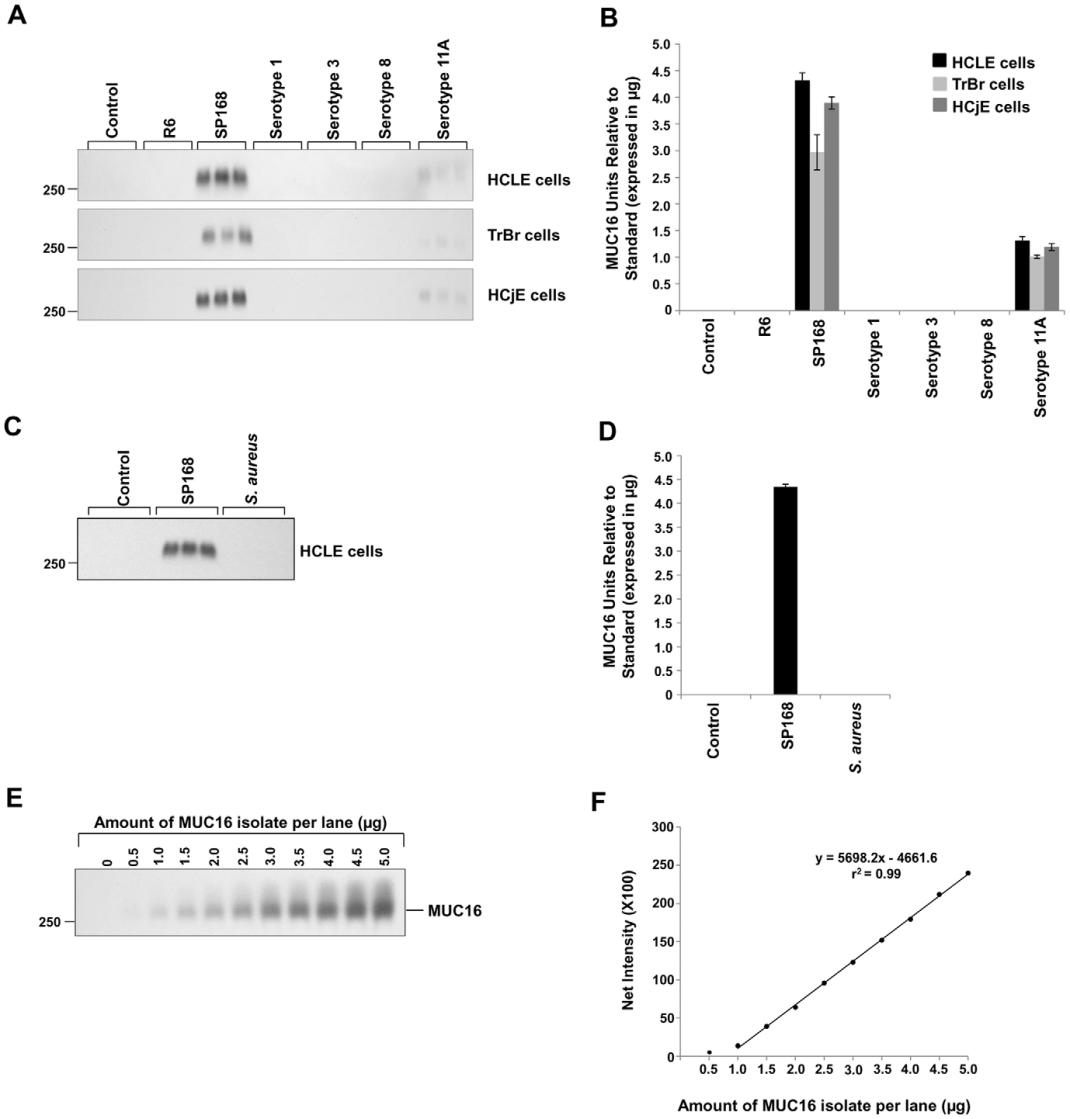
Epithelial MUC16 release by growth culture filtrates from different *S. pneumoniae* species and *S. aureus*. **A**) Western blots using M11, a MUC16 ectodomain-specific antibody, on culture supernatants of HCLE, TrBr, and HCjE cells after exposure to growth culture filtrates from *S. pneumoniae* strains R6 and SP168, and serotypes 1, 3, 8, and 11A. The 250 kDa standard marker is indicated on the left. **B**) Quantitative analyses of the western blots in (A) demonstrated that bacterial culture filtrates from only SP168 and serotype 11A induced release of the MUC16 ectodomain (n = 3). **C**) Western blot with the M11 antibody, on culture supernatants of HCLE cells after exposure to growth culture filtrates from *S. pneumoniae* strain SP168 and *S. aureus* ALC1435. **D**) Quantitative analysis of the western blot in (C) shows the inability of growth culture filtrate derived from *S. aureus* to induce MUC16 ectodomain shedding (n = 3). Data represent MUC16 units relative to the standard ± standard error of the mean (SEM). **E**) Western blot using the M11 antibody on known, increasing amounts of a partially purified MUC16 isolate. **F**) A standard curve that was generated by plotting band intensities that were in the linear range (E) vs known amounts of partially purified MUC16 protein.

**Figure 2:**
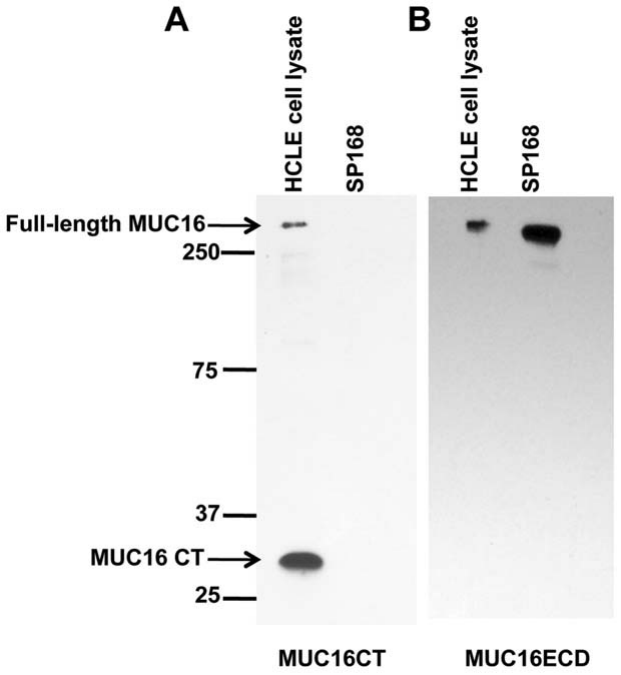
MUC16 released from epithelial surfaces by SP168 does not bind to a MUC16 cytoplasmic tail antibody indicating ectodomain release. HCLE cells were cultured with growth culture filtrate obtained from strain SP168 for 1 hour (SP168), and the resultant media was diluted 1∶3 with DMEM/F12, and subjected to a 10 kDa cutoff concentrator. HCLE cell lysate with intact full-length MUC16 (75 µg) was used as a positive control. Western blot of the resulting concentrate and cell lysate using: **A**) MUC16CT antibody that recognizes full-length MUC16 at >250 kDa and the cytoplasmic tail between 25–37 kDa, or **B**) MUC16 extracellular domain (ECD) antibody (M11). The MUC16CT antibody binds to the positive control cell lysate only (arrow), but not to the cell culture supernatants after exposure to SP168 (A). Conversely, M11 antibody binds to a band of appropriate molecular weight in culture supernatants as well as the cell lysate (B). These data indicate that the sheddase releases the MUC16 ectodomain into the cell culture media. Molecular weight standards are on the left in kDa.

To determine if clinical isolates of *S. pneumoniae*, both epidemic (strain SP168) and non-epidemic (serotypes 1, 3, 8 and 11A), as well as the laboratory strain R6, or whether the opportunistic pathogen *S. aureus* alter the MUC16 glycocalyx barrier in epithelial cells, human corneal (HCLE), conjunctival (HCjE), and tracheobronchial (TrBr) cells cultured for optimal mucin production [15] were treated with bacterial growth culture filtrates for 1 or 4 hours. Material released into the cell culture supernatants were analyzed by western blotting using a MUC16 ectodomain-recognizing antibody, M11 [16], as well as antibodies to MUC1 and MUC4, 214D4 [17], [18] and 8G7 [19], respectively. Western blot analyses indicated that only bacterial culture filtrates from strain SP168, which causes epidemic conjunctivitis, and serotype 11A, which has been associated with conjunctivitis [20] and pneumonia [21], induced MUC16 ectodomain release (Figure 1A, B). This MUC16 ectodomain release from HCLE, TrBr, and HCjE cells by the growth culture filtrate derived from strain SP168 was found to be higher than that induced by the filtrate from serotype 11A. Follow-up experiments have indicated that the *zmpC* gene in strain SP168, which is truncated, encodes a more potent zinc metalloproteinase (Menon B. B., *et al.*, manuscript in preparation). A small and variable amount of constitutive release of the MUC16 ectodomain was occasionally observed, especially from the ocular surface epithelial cell types (HCLE and HCjE). This phenomenon of constitutive release of the MUC16 ectodomain from ocular surface epithelial cells has been previously reported [22], [23], [24]; however, little is known about the innate mechanism(s) associated with constitutive MUC16 ectodomain release. Lack of binding of a MUC16 cytoplasmic tail antibody [23] to the released mucin demonstrates that the MUC16 in the culture media after treatment with the SP168 culture filtrate is a result of ectodomain release (Figure 2). No release of MUC16 ectodomain from the epithelial cell surface was observed following treatment with filtrates obtained from serotypes 1, 3, 8 and the laboratory strain R6 (Figure 1A, B) or from *S. aureus* (Figure 1C, D). The induction of MUC16 shedding was selective, in that MAMs MUCs 1 and 4 were not released by growth culture filtrates derived from strain SP168 (Figure S1 A, B).

### The amount of MUC16 on the epithelial cell surface is significantly reduced by the SP168 growth culture filtrate

**Figure 3:**
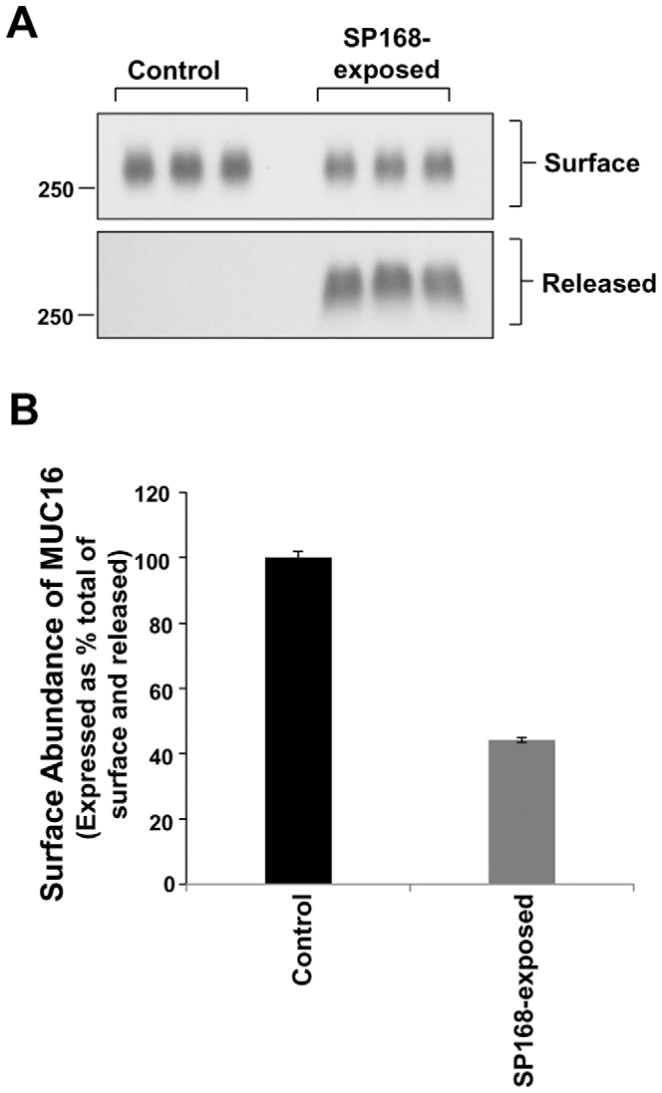
Apical cell surface abundance of MUC16 is reduced following treatment of epithelia with SP168 growth culture filtrate. Apical membrane levels of MUC16 were determined by cell surface biotinylation. **A**) Western blot analyses (using M11 antibody) of the amount of released and residual biotin-labeled surface MUC16 after exposure to medium only control or SP168 growth culture filtrate. **B**) Quantitative analyses of the western blot in (A) showing a reduction in the abundance of surface MUC16 following treatment with the SP168 culture filtrate (p<0.0001, Student's t-test, n = 3). Data for MUC16 surface abundance is expressed as a percentage of the total released plus surface MUC16 ± SEM.

The efficiency of the SP168 growth culture filtrate to remove the MUC16 ectodomain was determined by assaying the amount of residual MUC16 on the epithelial cell surface after treatment with the filtrate. As assay, epithelial cells were exposed to bacterial growth culture filtrates, followed by biotinylation of the epithelial cell surface proteins. Prior to biotinylation, cell culture supernatants were recovered from the HCLE cells treated with medium only as control or SP168 growth culture filtrate samples, for analysis of amount of released MUC16 protein. Western blot analyses were performed on samples of biotinylated cell surface MUC16 and released MUC16 ectodomain (Figure 3A). Treatment of epithelial cells with the SP168 growth culture filtrate resulted in a 56% decrease in surface abundance of MUC16 after 1 hour of exposure compared to total MUC16 levels (Figure 3B).

### The MUC16 sheddase secreted by *S. pneumoniae* is a zinc metalloproteinase, ZmpC

Preliminary experiments demonstrated that both heat inactivation and treatment with proteinase k, eliminate the sheddase activity in the SP168 growth culture filtrate, indicating that the sheddase is a protein (data not shown). Since *S. pneumoniae* strains are known to secrete metalloproteinases [25], SP168 growth culture filtrates were treated with metalloproteinase inhibitors GM6001 or TAPI-1 [26]. Filtrates treated with the metalloproteinase inhibitors showed a reduction of MUC16 ectodomain release compared to the medium only control (Figure S2). Thus, the protein of interest was predicted to be a secreted metalloproteinase.

**Figure 4:**
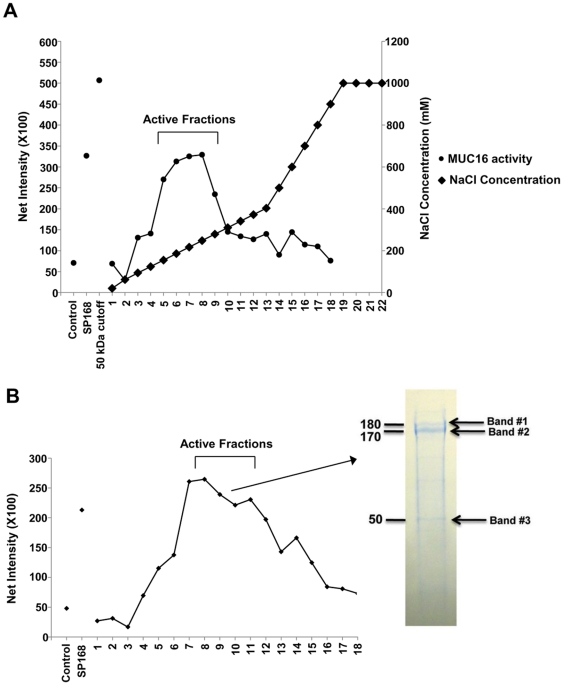
Isolation of the MUC16 sheddase and its identification as ZmpC. MUC16 sheddase-enriched fractions were isolated first by DEAE chromatography, with concentrations of sodium chloride eluent shown in the figure on the right (**A**), then by size exclusion chromatography (**B**). A sample from fraction 9 (indicated by an arrow) in B was separated by SDS-PAGE and stained using GelCode Blue stain. The 3 bands observed at ∼180 kDa, ∼170 kDa and ∼50 kDa were analyzed by mass spectrometry. Numbers on the left of the gel indicate molecular weight standards in kDa. The Y axes of graphs in (A) and (B) represent the net intensities corresponding to MUC16 signal on western blots. Constitutive MUC16 ectodomain release was observed in fractions whose net intensities correspond to that of the control. The mechanism(s) associated with constitutive MUC16 ectodomain shedding remains unknown.

To identify the sheddase in the SP168 growth culture filtrate, conventional protein purification methods were used, including filtration with a 50 kDa cutoff concentrator, followed by DEAE anion exchange, then size exclusion chromatography (SEC) (Figure 4 A, B). Peak sheddase activity was found between fractions 5–9 and 7–12, collected from the DEAE column and from SEC, respectively; the activity was determined by exposing HCLE cells to the different fractions that had been separated by chromatography and monitoring the fractions that induced maximal MUC16 ectodomain release. Of the fractions that exhibited peak MUC16 sheddase activity, fraction 9 of the SEC showed the maximum amount of total protein and was thus separated by SDS-PAGE and stained with GelCode blue. Three distinct bands, observed at ∼180 kDa, ∼170 kDa and ∼50 kDa (Figure 4B), were subjected to mass spectrometric analysis, which identified them as ZmpC (Table 1). The different molecular weights observed on the gel may represent cleavage products of ZmpC, as previously suggested by Chen *et al.* 2007 [26].

**Table 1:** Mass spectroscopic analysis of the purified SP168 sheddase identified as metalloproteinase ZmpC.

Band number	Protein	Peptide matches	% sequence coverage
1 ∼180 kDa	Zinc metalloproteinase ZmpC	126	59.8%
	Immunoglobulin A1 protease	13	7.3%
	Immunoglobulin A1	11	7.1%
2 ∼170 kDa	Zinc metalloproteinase ZmpC	141	62.2%
	Immunoglobulin A1 protease	15	7.3%
	Immunoglobulin A1 protease	10	6.0%
3 ∼50 kDa	Zinc metalloproteinase ZmpC	66	42.7%
	Glucose-6-phosphate isomerase	26	45.4%
	Aminopeptidase C	21	41.4%

### The SP168 deletion mutant (*zmpC*Δ) fails to induce MUC16 shedding in epithelial cells

**Figure 5:**
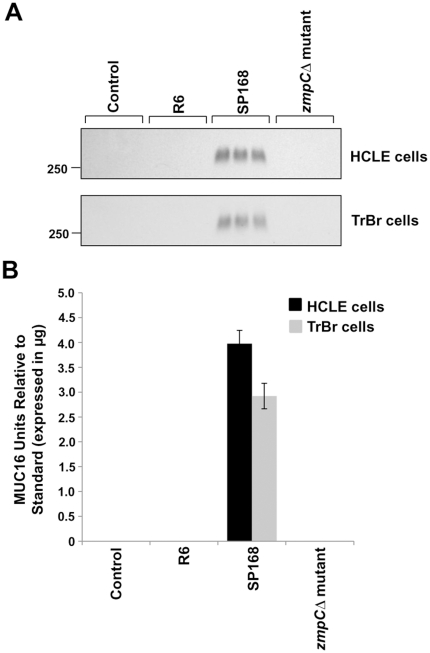
Epithelial MUC16 ectodomain release is induced by growth culture filtrate from SP168 but not from the SP168 *zmpCΔ* mutant. **A**) Western blots using M11 antibody comparing the amount of shed MUC16 in cell culture supernatants recovered after exposure of stratified HCLE and TrBr cells to bacterial growth culture filtrates from strains R6, wild type SP168 and the SP168 *zmpCΔ* mutant. **B**) Quantitative analyses of western blots in (A) show that the filtrate derived from the SP168 *zmpCΔ* mutant is unable to induce MUC16 ectodomain shedding in HCLE cells as well as in TrBr cells (n = 3). All data are represented as MUC16 units relative to the standard ± SEM.

**Figure 6:**
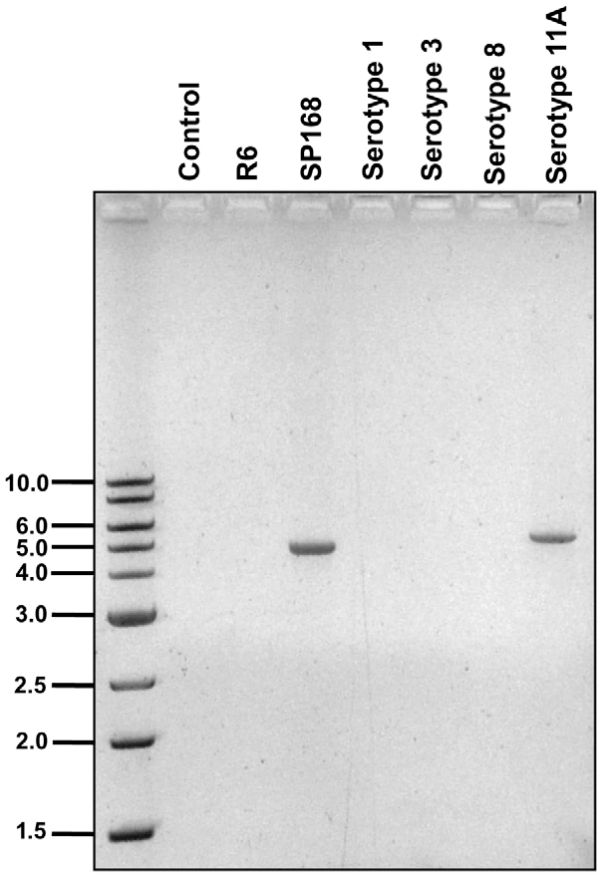
*zmpC* is present only in certain strains of *S. pneumoniae*. Genomic DNA isolated from all strains of *S. pneumoniae* under study was subjected to polymerase chain reaction to screen for the *zmpC* gene. Amplified products were separated on a 1% agarose/TBE gel. Molecular weight markers are represented in kilobase pairs (kbp). Products corresponding to the bands observed at ∼5 kbp were sequenced at the University of Maine DNA Sequencing Facility and identified as *zmpC*. Data indicated that the *zmpC* gene was present in *S. pneumoniae* strain SP168 and serotype 11A, the only strains exhibiting MUC16 sheddase activity. *zmpC* from *S. pneumoniae* strain SP168 was found to be 723 bp shorter than that of serotype 11A. The two sequences are 86.5% identical.

To verify that ZmpC is the MUC16 sheddase, a mutant strain was constructed from SP168 in which the *zmpC* gene was deleted and replaced with an erythromycin resistance cassette [27]. Epithelial cells were treated with culture medium, which served as control, or bacterial growth culture filtrates obtained from strains R6, which lacks the *zmpC* gene [28], wild type SP168, or its *zmpC*Δ mutant. In striking contrast to MUC16 shedding following treatment with wild type SP168, epithelial cultures treated with the growth culture filtrate obtained from the *zmpC*Δ mutant resulted in no MUC16 ectodomain release (Figure 5 A, B). These results confirm findings from mass spectrometric analysis, which indicated ZmpC as the MUC16 sheddase. Further evidence that ZmpC is indeed the MUC16 sheddase came from the finding that only those *S. pneumoniae* serotypes containing the *zmpC* gene induced MUC16 ectodomain shedding (Figures 1A and 6).

### Treatment of epithelial cells with the ZmpC-containing SP168 growth culture filtrate compromises the glycocalyx barrier

**Figure 7:**
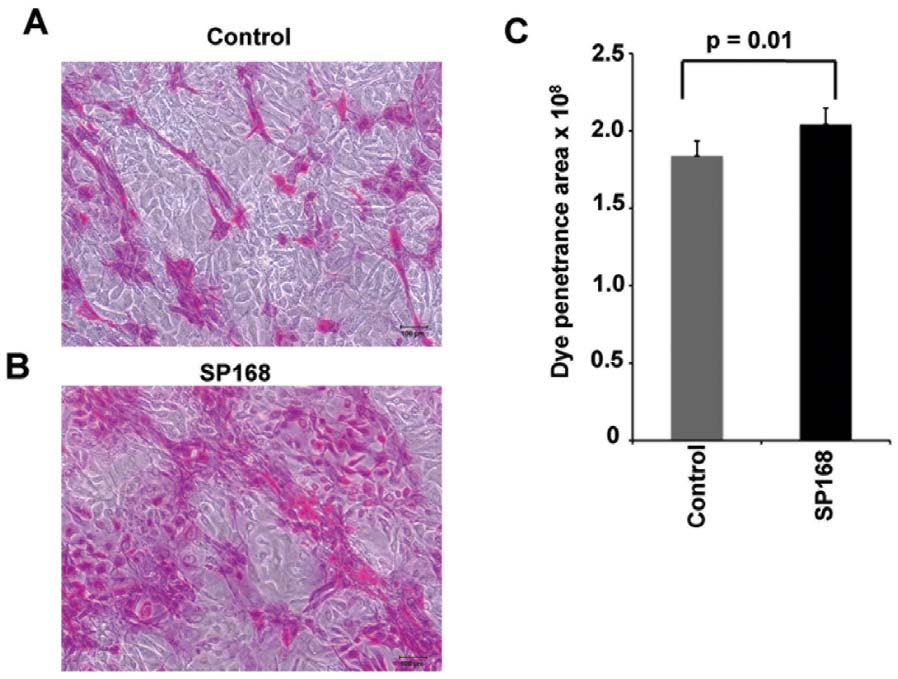
Rose bengal penetrance into HCLE cells increases after SP168 growth culture filtrate treatment. Brightfield micrographs were taken of HCLE cells stained with rose bengal dye following exposure to (**A**) medium only control or (**B**) ZmpC-containing growth culture filtrate obtained from strain SP168. Areas of cells with dye penetrance (p = 0.01, Mann-Whitney test, n = 55) were quantified using ImageJ software (**C**). Scale bar = 100 µm. These data indicate that the barrier efficiency of epithelial cells after exposure to ZmpC decreases due to loss of MUC16 ectodomain. Increased dye penetrance has been shown previously with siRNA knockdown of MUC16 [3].

Degree of penetrance of rose bengal, an anionic dye, is an established method of assaying epithelial barrier function [3], [14], [29]. It has previously been demonstrated that corneal epithelial cells exclude this dye [14], and that siRNA knockdown of MUC16 in these cells leads to increased uptake of the dye that in turn is associated with increased pathogen adherence [3]. It has also been reported that abrogation of O-glycosylation of MAMs in the glycocalyx and their association to galectin-3 lead to the loss of barrier function, as indicated by rose bengal dye penetrance [13]. To test if ZmpC-induced removal of MUC16 from the corneal epithelial surface results in loss of glycocalyx barrier function, HCLE cells were cultured with growth culture filtrates derived from strain SP168 for 4 hours, followed by staining of the epithelial cell surface with rose bengal dye. HCLE cells treated with the SP168-derived growth filtrate exhibited a statistically significant 20% increase in rose bengal dye penetrance in comparison to the control medium only-treated HCLE cells (Figure 7). These results indicate that removal of the MUC16 ectodomain by ZmpC diminishes glycocalyx barrier function.

### Strain SP168 invades stratified epithelial cells more efficiently than its isogenic *zmpC* deletion mutant (*zmpC*Δ)

**Figure 8:**
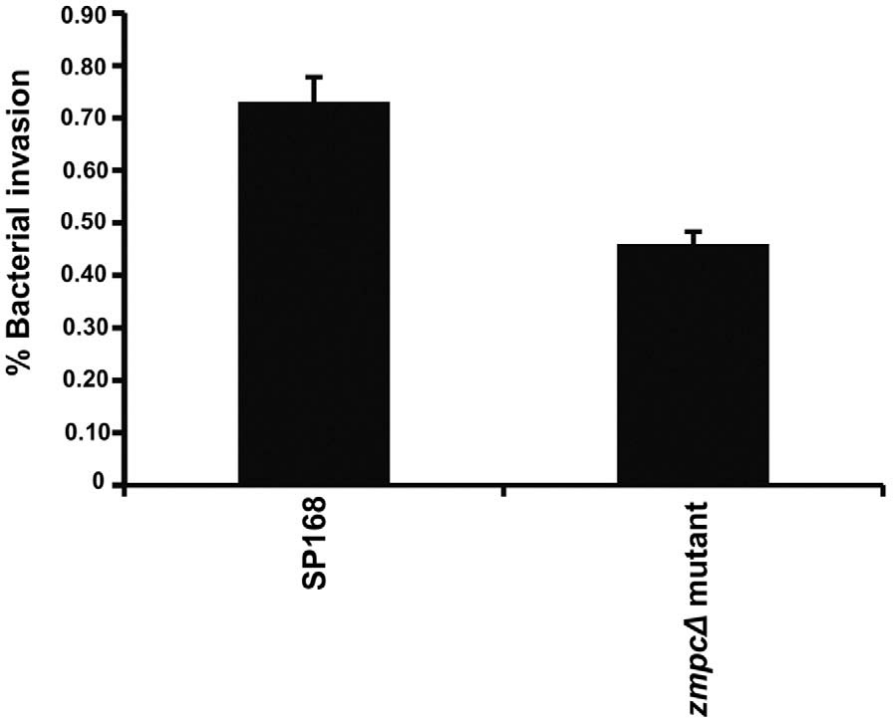
*S. pneumonia*e strain SP168 invades epithelial cells more efficiently than its isogenic *zmpC* Δ** mutant.** HCLE cells were incubated with SP168 and *zmpC*Δ mutant bacteria for 4 hours. Surface-bound bacteria were killed using an antibiotic protection assay. Quantitation of number of internalized bacteria was performed by serial dilutions of lysed cells and back-plating on blood agar plates. Data are expressed as a percentage of the number of bacteria recovered after the assay, divided by the initial inoculum number ± SEM (p<0.0001, unpaired t-test, n = 6).

To determine if the epidemic conjunctivitis-causing bacterium SP168 is able to invade the epithelial cells, a bacterial internalization assay was performed. Epithelial cells grown for optimal mucin production were cultured with SP168 bacteria or the SP168 *zmpC*Δ mutant for 4 hours. An antibiotic protection assay was used to determine the numbers of internalized bacteria [30]. A 2-fold, statistically significant difference was observed between the number of internalized bacteria with the SP168 strain compared to the *zmpC*Δ mutant (Figure 8). These data indicate that SP168 can more efficiently invade corneal epithelial cells as a result of exposure to the bacteria. Since cell invasion is a well-established process of infection, these experiments indicate that a loss in the levels of apical MUC16 on the epithelial barrier enhances internalization, thus infection.

## Discussion

In order for disease-causing, non opportunistic bacteria, to infect mucosal tissues, they must cross the MAM glycocalyx barrier that normally prevents adherence of opportunistic bacteria to epithelial surfaces [3], [11], [12]. Data provided herein describe for the first time a potential virulence mechanism that is utilized by a non opportunistic, infection-causing bacterium, *S. pneumoniae*, to compromise the MAM glycocalyx barrier to gain entry into the epithelium. We have identified and confirmed that a zinc metalloproteinase, ZmpC, secreted by certain virulent strains of *S. pneumoniae*, directly targets and induces ectodomain shedding of MUC16, a crucial defense component of the epithelial MAM glycocalyx barrier.

Bacterial extracellular zinc metalloproteinases have long been regarded as important contributors to the virulence of several pathogens [31]. *S. pneumoniae* secretes three zinc metalloproteinases including IgA1, ZmpB, and ZmpC [25]. These metalloproteinases contain the canonical zinc-binding motif HEXXH [31] and the highly conserved, prototypical LPXTG sequence of gram-positive cell surface proteins [32]. Intriguingly, they share very little homology at the primary amino acid sequence level. Of the three metalloproteinases, IgA1 has been the most extensively studied enzyme, and its role in pathogen virulence has been well documented [33], [34]. While the biological role of ZmpB remains unknown, ZmpC has been demonstrated to cleave the matrix metalloproteinase MMP-9 and contribute to streptococcal virulence in experimentally induced pneumonia [35], but the mechanism of its action remains unclear. ZmpC has also been shown to cause shedding of syndecan-1 from murine mammary gland epithelial cells [26]; however, the mammary gland epithelial surface does not represent a site easily accessible to mucosal infection-causing pathogens.

In the present study, a novel function for ZmpC has been discovered, in that the enzyme induces ectodomain shedding of the MAM MUC16 from epithelial cell surfaces – a finding supported by multiple lines of investigation. Using protein purification methods and mass spectrometric analyses, the enzyme in the SP168 growth culture filtrate that induces MUC16 ectodomain shedding from epithelial surfaces was identified as ZmpC. Furthermore, we showed that a *zmpC* deletion mutant (*zmpCΔ*), isogenic to strain SP168, is not capable of inducing MUC16 ectodomain shedding from epithelial surfaces. Finally, we found that only those strains of *S. pneumoniae* that induce MUC16 ectodomain shedding in HCLE, HCjE, and TrBr cells contain the *zmpC* gene. Demonstration that ZmpC is a sheddase specific for MUC16 may be most relevant to its potential virulence factor activity in pneumonia and conjunctivitis, since the tracheobronchial and ocular surface epithelia express high levels of the MAMs on their mucosal surfaces [15], [36].

MAMs, which form a major component of the glycocalyx on the apical surfaces of mucosal epithelia, where they can extend up to 500 nm from the epithelial surface [8], have been demonstrated to provide a barrier to pathogen and cell adherence [3], [11], [12], [37], [38]. Removal of the MAM MUC16 from epithelial surfaces by ZmpC secreted by certain strains of *S. pneumoniae* abrogates barrier function, as indicated by penetrance of rose bengal dye normally excluded from the epithelial cells [3], [14] and, importantly, internalization of the bacterium into the epithelial cell. Moreover, it has been previously shown that *S. pneumoniae* strain SP168 adheres to epithelial cells under culture conditions that do not promote mucin expression [37]. Taken together, these data suggest that epidemic infection-causing bacteria have the potential to manipulate the MAM barrier to gain access to epithelial surfaces.

Definitive proof that ZmpC is a virulence factor for conjunctivitis and pneumonia *in vivo* through its sheddase activity for MUC16, awaits the development of an appropriate animal model. Development of such a model is complicated by the fact that, while there are MAM homologues in the mouse, their size, glycosylation characteristics and, most importantly, their epithelial distribution is quite different than in human. For example, unlike in the human, Muc16 is not found in mouse ocular surface or reproductive tract epithelia [39]. While MUC16 is present in both mouse and human tracheobronchial epithelia, the mucins' structures vary dramatically, with a 258 aa length and single SEA module in the mouse, as compared to a 22,152 aa length and 56 SEA modules in human [40]. Moreover, MUC16's role in glycocalyx barrier formation in the mouse is not known. Perhaps the well-known cross species differences in susceptibility to bacterial infections [41] is influenced by the variability in character of glycocalyx membrane mucins among species.

It should be noted that Oggioni *et al.* have demonstrated that a *zmpC*Δ pneumococcus, constructed in strain FP23, shows a decrease in virulence in a mouse model for pneumonia [35]. These authors related the virulence to ZmpC-mediated cleavage of the proform of MMP-9 to activate the metalloproteinase. It is not known however, if MMP-9 plays a role in MUC16 ectodomain release in epithelial cells.

An alternative or additional explanation regarding consequences of MUC16 shedding from epithelial surfaces in response to ZmpC is that the released MUC16 ectodomain serves as a ‘decoy ligand’ [2], [11] for adhesins expressed on the cell surface of *S. pneumoniae*, preventing the pathogen from docking to the epithelial cell membrane. Following release of the MUC16 ectodomain, signaling by the MUC16 cytoplasmic tail could, in turn, potentially invoke an inflammatory response to clear the invading *S. pneumoniae* species. To date, this possibility has not been explored.

It remains to be seen if membrane mucin sheddases secreted by bacteria are a common virulence factor used by other disease-causing, non opportunistic bacteria. Since the repertoire of MAMs expressed at different mucosal surfaces varies (e.g., gut epithelia express MAMs MUCs 1, 3, 4, 11/12, and tracheobronchial/ocular surface/female reproductive tract epithelia express MUCs 1, 4 and 16), it would be interesting to determine if different membrane mucin sheddases act as virulence factors in gut infections than in MUC16-expressing epithelia. Moreover, it would be interesting to determine if the membrane mucin sheddases facilitate secondary infections by opportunistic pathogens.

**Figure 9:**
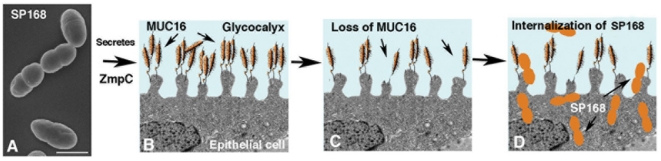
Diagram of the mechanism by which *S. pneumoniae* strain SP168 gains entry into epithelial cells. Scanning electron micrograph of *S. pneumoniae* strain SP168 (**A**), which secretes a zinc metalloproteinase, ZmpC, onto the apical surface of the epithelia (**B**), which has a glycocalyx rich in MUC16 transmembrane mucins that emanate from surface microridges (adapted from [47]). The secreted ZmpC induces MUC16 ectodomain release, causing a decrease in amount of MUC16 on the apical surface of the epithelium (**C**), that compromises the glycocalyx barrier integrity. *S. pneumoniae*, depicted in orange, is thus able to invade the epithelial cells due to the loss of MUC16 (**D**).

In summary, this is the first demonstration that metalloproteinases secreted by non opportunistic bacteria can induce ectodomain release of membrane-associated mucins. Loss of the membrane mucin barrier compromises epithelial integrity and promotes internalization of the bacteria to facilitate infection (Figure 9).

## Materials and Methods

### Ethics statement

As described previously for development and characterization of the telomerase-transformed human corneal (HCLE) and human conjunctival (HCjE) epithelial cell lines used in this study [15], [42], human corneal epithelial cells were derived from human corneoscleral rims provided by Roger Steinert and Ann Bajarat of Ophthalmic Consultants of Boston. Human conjunctival biopsy specimens with normal ocular surfaces were provided by C. Steven Foster of the Massachusetts Eye and Ear Infirmary. These samples were obtained without patient identifiers as discarded tissue post surgery.

Primary cultures of human tracheobronchial epithelial cells were commercially obtained, without patient identifiers from Clonetics (Lonza; Walkersville, MD). Research involving the study of existing data, documents, records, pathological specimens, or diagnostic specimens (source of tracheobronchial epithelial cells), if these sources are publicly available and used in such a manner that subjects cannot be identified, directly or through identifiers linked to the subjects, is considered exempt from Institutional Review Board (IRB) under HHS regulations at 45 CFR 46.101(b)(4).

### Culture of human ocular surface cell lines

Telomerase immortalized human corneal (HCLE) and conjunctival (HCjE) epithelial cells were cultured in keratinocyte serum-free medium (Invitrogen, Carlsbad, CA) until confluence, then switched to Dulbecco's Modified Eagle Medium: Nutrient Mixture F-12 (DMEM/F12; Cellgro, Manassas, VA) supplemented with 10% calf serum and 10 ng/mL epidermal growth factor (EGF) for 7 days to promote differentiation, stratification and optimal membrane mucin production [15].

### Derivation and culture of tracheobronchial epithelial cell line

Primary cultures of human tracheobronchial epithelial cells obtained from Clonetics (Lonza) were immortalized by expression of hTERT and abrogation of p16 and p53 function. Full details of the techniques used for derivation of the cell line have been reported [15], [42].

Tracheobronchial epithelial cells were cultured and grown to confluence in Bronchial Epithelial Cell Basal Medium (Clonetics, Lonza) containing manufacturer recommended growth supplements and then switched to 10% serum+10 ng/mL EGF-containing DMEM/F12 medium for 7 days to promote optimal mucin production. When grown on plastic, the tracheobronchial cells express MUCs 1, 4 and 16 on the apical epithelial surface (data not shown).

### Bacterial growth culture filtrates

*S. pneumoniae* serotypes 1, 3, 8, 11A, R6, and strain SP168 were obtained from the Centers for Disease Control and Prevention (Atlanta, GA) and were routinely streaked and grown on 5% sheep blood agar plates (Becton Dickinson; Franklin Lakes, NJ) [37]. Individual colonies from plates were inoculated in Todd-Hewitt (TH) broth containing 0.5% w/v yeast extract and grown to an OD_595_ of 0.22. *Staphylococcus aureus* strain ALC1435, a *sarP1::gfp* derivative of RN6390 (gift of Dr. Ambrose L. Cheung, Dartmouth College; Hanover, NH), was grown in brain heart infusion medium (BHI; Research Products International; Mt. Prospect, IL) to an OD_595_ of 0.4. All liquid cultures were grown under static conditions, and all incubations were at 37°C with 5% CO_2_. To obtain bacterial growth culture filtrates, liquid cultures grown to a density as indicated above were centrifuged at 4000× g for 10 minutes at RT. The resulting supernatants were filtered through a 0.22 µm filter and stored at −80°C until the filtrates were tested for MUC16 sheddase activity. The growth culture filtrates were stable for at least 2 years at −80°C without freeze-thaw.

### Western blot analysis of membrane mucins in epithelial culture medium after treatment of epithelial cell cultures with bacterial growth culture filtrates

Cells were cultured in 24 well plates (1.9 cm^2^/well) or 6 well plates (9.5 cm^2^/well). Cells were washed three times with 1 mL of DMEM/F12 media prior to treatment with bacterial growth culture filtrates. Epithelial cell cultures were treated with bacterial growth culture filtrates or Todd-Hewitt broth (medium only control) pre-diluted 1∶1 with DMEM/F12 for 1 or 4 hours at 37°C with 5% CO_2_. Cell culture supernatants were recovered after filtration using a 0.22 µm filter. 500 µL of supernatants were concentrated using a 10 kDa cutoff concentrator (Millipore; Cork, Ireland). The retention volume was 25–30 µL. The 25–30 µL sample, reduced in Laemmli sample buffer, was loaded on a gel and analyzed by SDS-agarose electrophoresis followed by western blotting for MUC16 release using the M11 antibody [16] (Neomarkers; Fremont, CA), which recognizes the ectodomain region of the molecule and with horseradish peroxidase-conjugated goat anti-mouse IgG_1_ (Santa Cruz Biotechnology, sc-2969; Santa Cruz, CA) as the secondary antibody. A MUC16 cytoplasmic tail-specific antibody, MUC16 CT [23] was used to determine if the released mucin lacked its cytoplasmic tail. The secondary antibody used in this blotting procedure was horseradish peroxidase-conjugated goat anti-rabbit IgG (Santa Cruz Biotechnology, sc-2054). Western blot analyses for MUC1 and MUC4 ectodomain release were performed using the 214D4 antibody (Upstate Cell Signaling Technologies; Lake Placid, NY) and 8G7 antibody (a gift from Dr. Surinder K. Batra; University of Nebraska Medical Center) specific to the MUC1 and MUC4 ectodomains, respectively. Horseradish peroxidase-conjugated goat anti-mouse IgG_1_ (Santa Cruz Biotechnology, sc-2969) was used as the secondary antibody. Western blots were developed using the SuperSignal West Femto Maximum Sensitivity Substrate (Thermo Scientific; Rockford, IL). Band intensities on blots were measured using the Kodak dS 1D digital science software, version 2.02, from Kodak Imaging systems (New Haven, CT).

Quantitation of relative amounts of MUC16 protein in samples was done by extrapolation from a standard curve. This curve was generated by running known amounts of a partially purified MUC16 isolate, ranging from 0 µg to 5.0 µg, in parallel with samples (Figure 1E, F). The partially pure fraction of MUC16, which was used for plotting the standard curve, was isolated from the ovarian adenocarcinoma cell line, NIH:OVCAR-3 (ATCC HTB-161) [43], using the protocol described by Schultes, B. C. *et al.* [44]. After separation of the proteins by SDS-agarose gel electrophoresis and subsequent western blotting, a plot was generated with band intensities on the *Y* axis and known amounts of partially purified MUC16 protein on the *X* axis. Data points that were within the linear range were used to generate the standard curve, which relates the amount of protein to signals on the blot. The slope-intercept form of the linear equation obtained from the standard curve was used to quantitate the relative amounts of MUC16 protein in samples. For instance, in Figure 1B, the amount of MUC16 protein released from HCLE, TrBr, and HCjE cells (Figure 1A) was calculated by substituting band intensities obtained from samples for y values in the equation, y = 5698.2x−4661.6 (Figure 1F).

### Surface protein biotinylation

Stratified HCLE cells grown for optimal mucin production, as described above, were treated with the SP168 growth culture filtrate or medium control for 1 hour. Biotinylation of cell surface proteins was performed as described [23]. Briefly, after the 1 hour treatment, the cells were incubated with 500 µg/mL sulfo-NHS-biotin (Pinpoint Cell Surface Protein Isolation kit, Pierce; Rockford, IL) for 30 minutes at 4°C. Cells were lysed and biotinylated cell surface proteins were isolated using NeutrAvidinTM gel. Biotinylated proteins were eluted using 50 mM dithiothreitol. The amount of surface biotinylated MUC16 remaining after treatment with the SP168 growth culture filtrate is represented as a percentage of the total cellular MUC16 (released MUC16 plus surface MUC16).

### Purification of the MUC16 sheddase from the SP168 growth culture filtrate

Initially, the SP168 growth culture filtrate (140 mL) was subjected to 50 kDa cutoff concentration (Millipore) and 150 µL was recovered as the retention volume. After testing for MUC16 sheddase activity on HCLE cells, 2 mg of protein from the concentrated sample was resuspended in 1 mL of buffer (20 mM Tris, 20 mM NaCl, pH 7.4) and subjected to anion exchange chromatography using a HiTrap DEAE FF 5 mL column (GE Healthcare; Piscataway, NJ). Fractions were collected using a 0.02–1 M NaCl gradient at a flow rate of 1 mL/minute. All fractions were tested for MUC16 sheddase activity on HCLE cells. Active fractions from 3 runs (protein concentration between 0.23–0.43 mg/mL) of anion exchange chromatography, found to elute between 150–300 mM NaCl, were pooled and subjected to a 10 kDa cut off concentrator. 0.8 mg of protein of the concentrated, pooled active fraction was diluted in 90 µL of elution buffer (50 mM Tris, 50 mM NaCl, pH 7.4) and loaded onto a 4% cross-linked, agarose bead (CL-4B) 10×30 cm column with a 25 mL bed volume. Fractions, eluted at a flow rate of 0.15 mL/minute, were tested for MUC16 sheddase activity. Both ion exchange and size exclusion chromatography were performed using a Dionex Bio-LC chromatography system (Sunnyvale, CA). Protein concentrations were determined using micro BCA analysis (Pierce) prior to and after each step of purification.

### Mass spectroscopic analyses

Fractions with peak activity from size exclusion chromatography were resolved by 10% SDS-PAGE and stained with GelCode Blue stain (Thermo Scientific). The 3 bands observed at ∼180 kDa, ∼170 kDa, and ∼50 kDa were excised and submitted for MALDI-TOF analyses to the TAPLIN mass spectroscopy facility, Harvard Medical School, Boston, MA.

### Polymerase chain reactions to amplify *zmpC*

Presence of the *zmpC* gene in the different strains of *S. pneumoniae* was determined by polymerase chain reaction (PCR). Primers corresponding to the 5′ and 3′ regions of *S. pneumoniae* TIGR4 *zmpC* (GenBank: AAK74260.1) were used. Primer sequences are listed in Table S1. Genomic DNA isolated from the different strains [45] served as templates in reactions. Amplified products were sequenced at the University of Maine DNA Sequencing Facility to confirm the identity of *zmpC*.

### Construction of the SP168 *zmpC* deletion mutant (*zmpC*Δ) mutant

The SP168 *zmpC*Δ mutant was constructed by replacing the *zmpC* gene of wild type SP168 with an erythromycin resistance cassette *(ermAM)*. Sequences that were identical to the 5′ upstream and 3′ downstream regions of *zmpC* were fused to the *ermAM* cassette on either side by overlap extension PCR to drive homologous recombination. The replacement fragment, at a concentration of 0.5 µg/mL, was used to transform SP168 cells that were made competent using competence stimulating peptide (CSP) [30], [46]. Transformants were selected on casitone+tryptone agar plates containing 0.25 µg/mL erythromycin [27]. The correct *zmpC*Δ mutant was confirmed by PCR. Primers used in the construction of the *zmpC*Δ mutant are listed in Table S1.

### Rose bengal dye penetrance assay for determining barrier integrity

Corneal epithelial cells (HCLE) were grown to stratification and optimal mucin expression as described above. Cells were treated with medium only control or SP168 growth culture filtrate for 4 hours. Cells were then washed 3 times with phosphate-buffered saline (PBS), pH 7.4, followed by incubation for 5 minutes with 0.1% w/v rose bengal dye (Acros Organics; Morris Plains, New Jersey) prepared in calcium/magnesium-free PBS. The dye was then aspirated and cell cultures were photographed [3]. ImageJ software (NIH) was used to quantify the areas of the cell cultures that incorporated the rose bengal dye.

### SP168 and *zmpC*Δ mutant internalization assay

Pneumococcal internalization assays were performed as described by Pracht, D. *et al.* [30]. Briefly, HCLE cells were grown to optimal mucin production and switched to antibiotic-free stratification medium for 24 hours prior to the internalization assay. Cells were then washed 3 times with DMEM/F12 medium; next SP168 or *zmpC*Δ bacteria grown to an OD_595_ of 0.05–0.1 in TH broth and diluted 1∶1 in DMEM/F12 medium were added. After incubation of the epithelial cultures for 4 hours with the bacteria at 37°C/5% CO_2_, unbound bacteria were washed away by rinsing the HCLE cell surface with DMEM/F12 medium. Extracellular bound bacteria were killed by incubation of HCLE cells with DMEM/F12 medium containing 100 µg/mL gentamicin and 100 U/mL penicillin G for 1 hour at 37°C. Cells were then washed 3 times with sterile 1× PBS and treated with 0.05% trypsin-EDTA for 10 minutes at 37°C/5% CO_2_. Internalized pneumococci were recovered by lysing the HCLE cells with 1% w/v saponin prepared in DMEM/F12 medium. Serial dilutions of epithelial cells containing internalized pneumococci were prepared and plated on 5% sheep blood agar plates. Following 18–24 hour incubation of the plates at 37°C/5% CO_2_, colony forming units corresponding to the pneumococci were counted. The number of the internalized bacteria is expressed as the number of pneumococci recovered from the antibiotic protection assay divided by the number of bacteria initially added to HCLE cells.

## Supporting Information

Figure S1 **HCLE cells do not release MUC1 and MUC4 ectodomain in response to** ***S. pneumoniae*** **growth culture filtrates.** Western blot analyses on culture supernatants of HCLE cells after exposure to growth culture filtrates from *S. pneumoniae* strain SP168 using the 214D4 antibody, which recognizes the MUC1 ectodomain (**A**) or with 8G7 antibody, which recognizes the MUC4 ectodomain (**B**). +Control = HCLE cell lysate, control = medium exposure only, SP168 = with SP168 growth culture filtrate.
(TIF)

Figure S2 **Treatment of the SP168 culture filtrate with matrix metalloproteinase inhibitors decreases its ability to induce MUC16 shedding from epithelial cells.** HCLE cells were cultured with either SP168-derived growth culture filtrates or medium only (control that had been previously treated with either 100 µM of the metalloproteinase inhibitors GM6001 or TAPI-1 or their vehicle (DMSO) for 1 hour at 37°C. Culture supernatants, analyzed by western blotting using the M11 antibody, showed a significant decrease in MUC16 shedding by the filtrate treated with GM6001 and TAPI-1. The Y axis of the graph represents the net intensities corresponding to MUC16 signal on western blots. Constitutive MUC16 ectodomain release was observed in the media of control conditions (i.e. Control, Control+DMSO, GM6001, and TAPI-1). The levels of MUC16 ectodomain release in the experimental conditions containing the metalloproteinase inhibitors (i.e. SP168+GM6001 and SP168+TAPI-1) were similar to those observed in the control conditions. The mechanism(s) associated with constitutive MUC16 ectodomain shedding remains unknown.
(TIF)

Table S1 Primers used to construct the SP168 *zmpC* deletion mutant.
(DOC)
