# Supplementary figures and images for: A Metalloproteinase Secreted by Streptococcus pneumoniae Removes Membrane Mucin MUC16 from the Epithelial Glycocalyx Barrier
Source: PLoS One. 2012 Mar 7;7(3):e32418. doi: 10.1371/journal.pone.0032418 (PMC3296694; doi:10.1371/journal.pone.0032418)

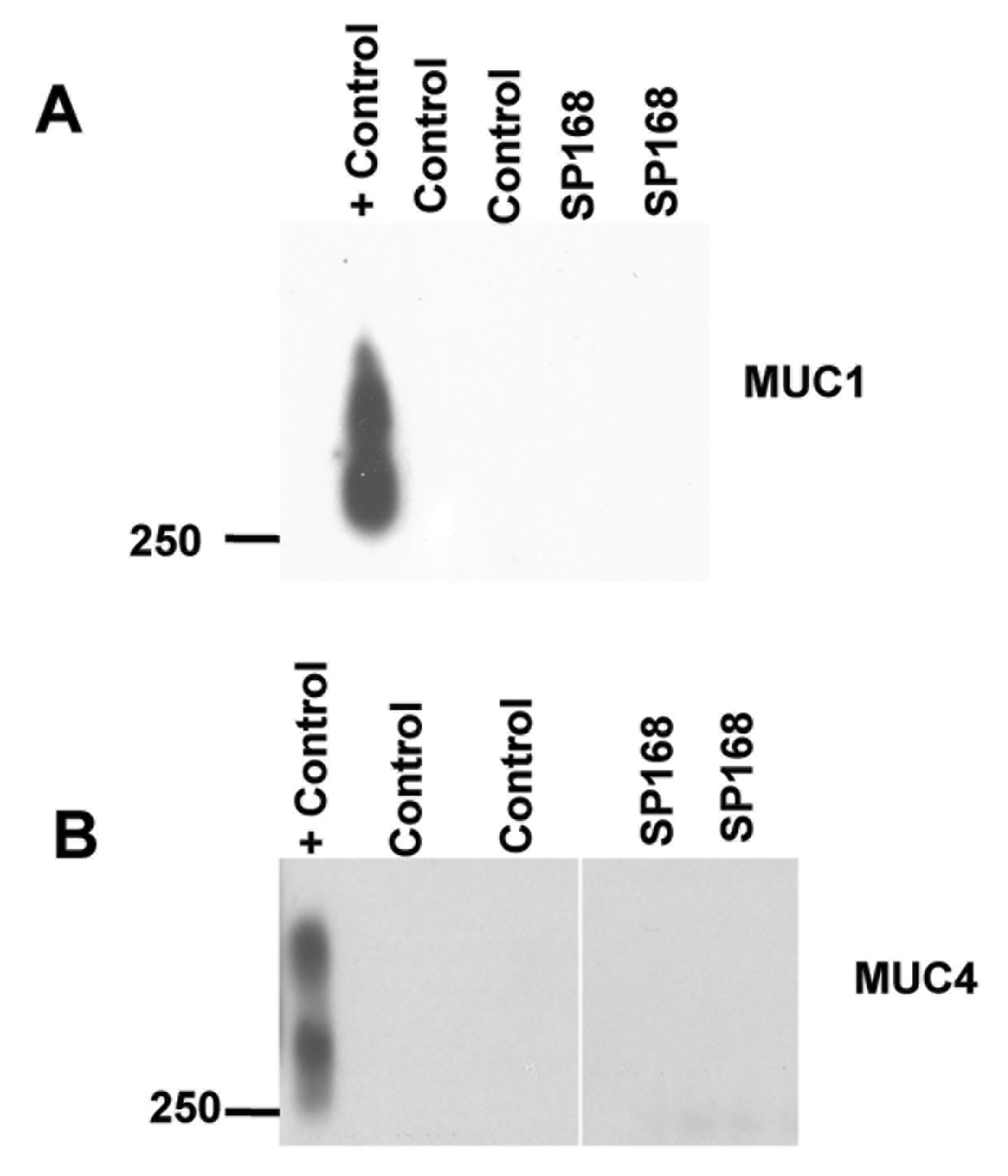

Supplement: Figure S1 — HCLE cells do not release MUC1 and MUC4 ectodomain in response to S. pneumoniae growth culture filtrates. Western blot analyses on culture supernatants of HCLE cells after exposure to growth culture filtrates from S. pneumoniae strain SP168 using the 214D4 antibody, which recognizes the MUC1 ectodomain (A) or with 8G7 antibody, which recognizes the MUC4 ectodomain (B). +Control = HCLE cell lysate, control = medium exposure only, SP168 = with SP168 growth culture filtrate. (TIF) [file pone.0032418.s001.tif]

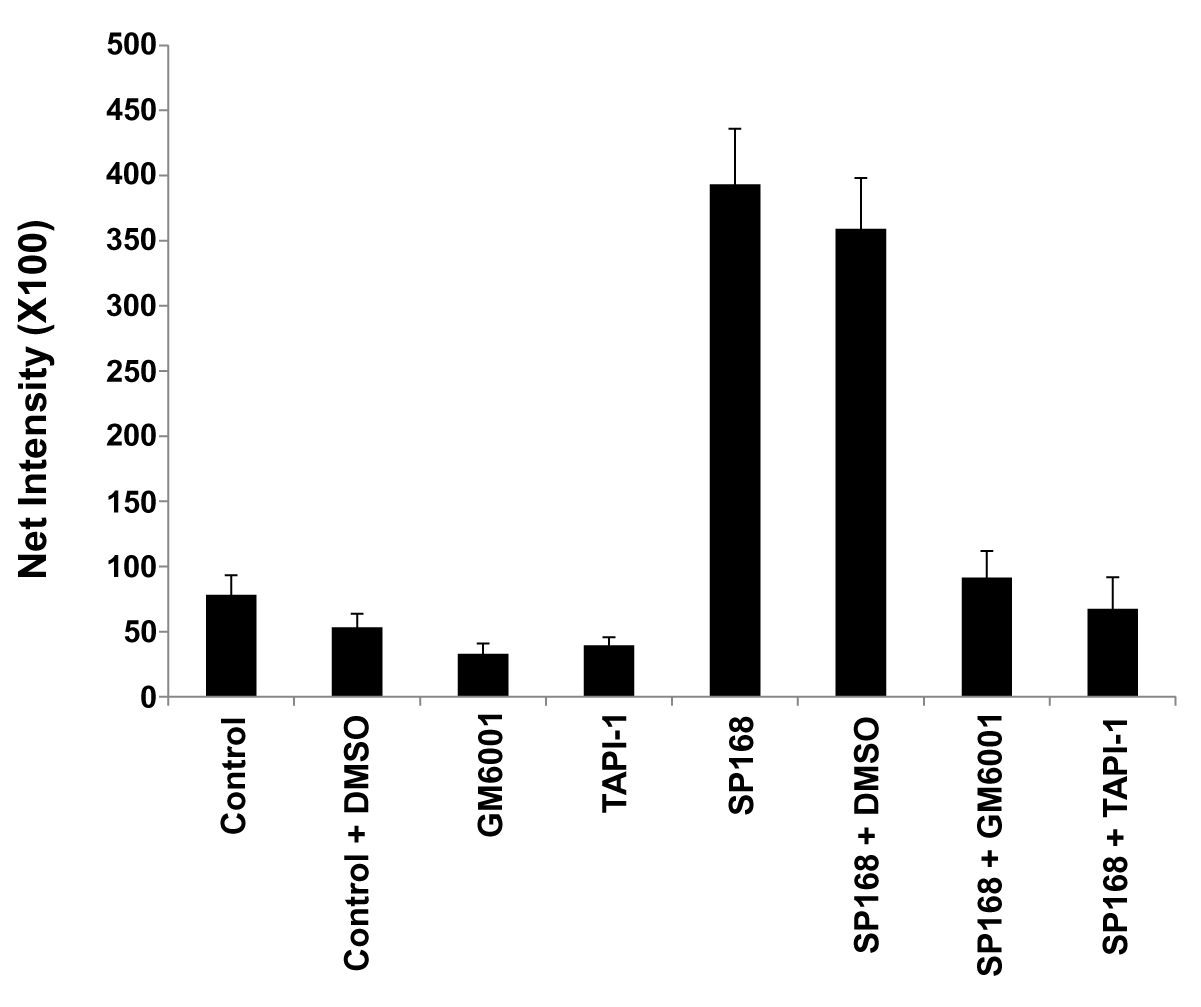

Supplement: Figure S2 — Treatment of the SP168 culture filtrate with matrix metalloproteinase inhibitors decreases its ability to induce MUC16 shedding from epithelial cells. HCLE cells were cultured with either SP168-derived growth culture filtrates or medium only (control that had been previously treated with either 100 µM of the metalloproteinase inhibitors GM6001 or TAPI-1 or their vehicle (DMSO) for 1 hour at 37°C. Culture supernatants, analyzed by western blotting using the M11 antibody, showed a significant decrease in MUC16 shedding by the filtrate treated with GM6001 and TAPI-1. The Y axis of the graph represents the net intensities corresponding to MUC16 signal on western blots. Constitutive MUC16 ectodomain release was observed in the media of control conditions (i.e. Control, Control+DMSO, GM6001, and TAPI-1). The levels of MUC16 ectodomain release in the experimental conditions containing the metalloproteinase inhibitors (i.e. SP168+GM6001 and SP168+TAPI-1) were similar to those observed in the control conditions. The mechanism(s) associated with constitutive MUC16 ectodomain shedding remains unknown. (TIF) [file pone.0032418.s002.tif]
