# Supplementary material for: A Metalloproteinase Secreted by Streptococcus pneumoniae Removes Membrane Mucin MUC16 from the Epithelial Glycocalyx Barrier
Source: PLoS One. 2012 Mar 7;7(3):e32418. doi: 10.1371/journal.pone.0032418 (PMC3296694; doi:10.1371/journal.pone.0032418)
Supplement: Table S1 — Primers used to construct the SP168 zmpC deletion mutant. (DOC) [file pone.0032418.s003.doc]

**Supplementary Table S1.**

**Primers used to construct the SP168 *zmpC* deletion mutant**

| **Primer name** | **Primer sequence (5’🡪3’)** |
| --- | --- |
| P1 | GATCCGGAATTCTTCAATCTCAGACTTTTCTATTATGAGCTAATATTTTATAGTATATTAAAAGCATAATCGGTAATCTA |
| P2 | ATTTTAAGATACAAATCAAACAAATTTTGGGCCCGGGGCGCGCCATTAACCTCGCTTTTTCTACATTCTTTATACCAATC |
| P3 | GATTGGTATAAAGAATGTAGAAAAAGCGAGGTTAATGGCGCGCCCCGGGCCCAAAATTTGTTTGATTTGTATCTTAAAAT |
| P4 | ACAAGAAACACTACTCAACAATGAAACTCTACAATCGGCCGGCCAGTCGGCAGCGACTCATAGAATTATTTCCTCCCGTT |
| P5 | AACGGGAGGAAATAATTCTATGAGTCGCTGCCGACTGGCCGGCCGATTGTAGAGTTTCATTGTTGAGTAGTGTTTCTTGT |
| P6 | CTGCAGCTCGAGAGCCCTAACACCGTTTGATAAAACTGTCTGGACTTTCGACTATCCTTGACAGAAAGATGAATATGCCC |

**Primers used to amplify *zmpC***

| **Primer name** | **Primer sequence (5’🡪3’)** |
| --- | --- |
| P7 | ATAT*ACTAGT*ATGAGCCGAAAAAGCATTGGTGAGAAACGC |
| P8 | CGCG*AAGCTT*TTATTTATATATAGAATTTCTAAAGTCATC |

Underlined sequences in primers P7 and P8 bear homology to the 5’ and 3’ regions of *S. pneumoniae* TIGR4 *zmpC* (GenBank: AAK74260.1), respectively. Italicized sequences represent SpeI and HindIII restriction enzyme sites.
